# Appendiceal abscess: pathologic definitions and a diagnostic framework—SIRC/SICUT consensus

**DOI:** 10.1007/s00384-026-05174-y

**Published:** 2026-06-15

**Authors:** Roberto Cirocchi, Gioia Brachini, Giovanni Alemanno, Massimiliano Allegritti, Lana Al-Sabe, Gabriele Anania, Nikolaos-Achilleas Arkoudis, Marco Assenza, Paolo Aurello, Francesco Barberini, Guido Bellezza, Maria Irene Bellini, Alan Biloslavo, Carlo Boselli, Francesco Brucchi, Paolo Bruzzone, Diletta Cassini, Isaac Cheruiyot, Bruno Cirillo, Federico Coccolini, Mario Corona, Piero Covarelli, Valerio Cozza, Daniele Crocetti, Justin Davies, Gunjan Desai, Saurabh Dixit, Alessandro Spizzirri, Vincenzo Napolitano, Daniele Giuliani, Alessio Giordano, Akinfemi Akingboye, Weihua Gong, Salvatore Guarino, Ahmed H. I. Helmy, Sara Lauricella, Augusto Lauro, Gianluca Mascianà, Matteo Matteucci, Alessandra Panarese, Mauro Podda, Georgi Popivanov, Paolo Prosperi, Camilo Ramírez-Giraldo, Dario Tartaglia, Jacopo Tesei, Luca Tomassini, Samuele Vaccari, Vipul D. Yagnik, Xiaonan Xiang, Mauro Zago, Giuseppe Nigri, Enrico Fiori, Antonia Rizzuto, Paolo Sapienza, Andrea Mingoli, Vito D’Andrea, Antonio Pesce

**Affiliations:** 1 Department of Medicine and Surgery, University of Perugia, 06123 Perugia, Italy; 2 Department of Surgery, Policlinico Umberto I, Sapienza University of Rome, 00161 Rome, Italy; 3 Emergency Surgery Unit, Careggi University Hospital, 50139 Florence, Italy; 4 Interventional Radiology Unit, Santa Maria Hospital, 05100 Terni, Italy; 5 Department of General Surgery, School of Medicine, The University of Jordan, Amman, 11942 Jordan; 6 Department of Surgery, AUSL of Ferrara, 44121 Ferrara, Italy; 7 Research Unit of Radiology and Medical Imaging, National and Kapodistrian University of Athens, 11528 Athens, Greece; 8 Section of Anatomic Pathology and Histology, Department of Medicine and Surgery, University of Perugia, 06156 Perugia, Italy; 9 Emergency Surgery Section, Cattinara University Hospital, 34149 Trieste, Italy; 10 Division of General Surgery, IRCCS Istituto Auxologico Italiano, 20145 Milan, Italy; 11 Unit of General Surgery, Sesto San Giovanni Hospital, ASST Nord Milano, 20099 Sesto San Giovanni, Italy; 12 Faculty of Health Sciences, University of Nairobi, P.O. Box 30197-00100, Nairobi, Kenya; 13 General and Emergency Surgery, University Hospital of Pisa, 56124 Pisa, Italy; 14 Department of Radiology, Policlinico Umberto I, Sapienza University of Rome, 00161 Rome, Italy; 15 Emergency Surgery and Trauma Unit, Fondazione Policlinico Universitario A. Gemelli IRCCS, 00168 Rome, Italy; 16 Cambridge Colorectal Unit, Addenbrooke’s Hospital, Cambridge University Hospitals NHS Foundation Trust, Cambridge, CB2 0QQ UK; 17 Lilavati Hospital and Research Centre, Mumbai, 400050 Maharashtra India; 18 Highland Care Multispeciality Hospital, New Delhi, 110059 India; 19 Department of General Surgery, Santa Maria Hospital, 05100 Terni, Italy; 20 The Dudley Group NHS Foundation Trust, Dudley DY1 2HQ, United Kingdom and College of Health and Life Science, Aston University Birmingham, Birmingham, UK; 21 Department of Surgery, Second Affiliated Hospital, Zhejiang University School of Medicine, Hangzhou, 310009 China; 22 Department of General Surgery, IRCCS MultiMedica, 20099 Sesto San Giovanni, Italy; 23 Department of General Surgery, School of Medicine, New Giza University, Giza, 12585 Egypt; 24 Department of Surgery, Fondazione IRCCS Istituto Nazionale Dei Tumori, 20133 Milan, Italy; 25 Colorectal Surgery Clinical and Research Unit, Surgery Center, Fondazione Policlinico Universitario Campus Bio-Medico, Campus Bio-Medico University of Rome, 00128 Rome, Italy; 26 Department of Medicine and General Surgery, University of Milan, 20145 Milan, Italy; 27 Department of Biotechnological and Applied Clinical Sciences, University of L’Aquila, 67100 L’Aquila, Italy; 28 Department of Surgical Sciences, University of Cagliari, 09124 Cagliari, Italy; 29 Department of Surgery, Military Medical Academy, 1606 Sofia, Bulgaria; 30 Hospital Universitario Mayor–Méderi, 111321 Bogotá, Colombia; 31 School of Advanced Studies, University of Camerino, 62032 Camerino, Italy; 32 Emergency Surgery Unit, Department of General Surgery, Bentivoglio Hospital, AUSL Bologna, 40010 Bentivoglio, Italy; 33 Department of Surgery, Banas Medical College and Research Institute, Palanpur, 385001 Gujarat India; 34 Department of Colorectal Surgery, Sir Run Run Shaw Hospital, Zhejiang University School of Medicine, Hangzhou, 310016 China; 35 Department of General Surgery, A. Manzoni Hospital, Lecco, Italy; 36 Department of Medical and Surgical Sciences, Magna Graecia University of Catanzaro, Catanzaro, Italy

**Keywords:** Appendiceal abscess, Acute appendicitis, Consensus; Delphi technique, Diagnostic imaging, Computed tomography, Pathologic classification

## Abstract

**Background:**

Appendiceal abscesses are a heterogeneous manifestation of acute appendicitis, with diverse pathological substrates and imaging phenotypes. The lack of standardized definitions hampers cross-study comparability and consistent diagnostic pathways.

**Methods:**

The Italian Society of Surgical Research (SIRC) and the Italian Society of Emergency Surgery and Trauma (SICUT) conducted a four-round modified Delphi, culminating in an in-person consensus conference (Rome, November 6, 2025). A multidisciplinary panel refined the statements on pathological definitions, classifications, and imaging correlates.

**Results:**

Consensus was reached on 22 statements. Part 1 reports seven statements that delineate the spectrum from uncomplicated to complicated appendicitis (phlegmonous, gangrenous, and perforated) and distinguishes appendiceal abscesses from appendiceal masses. An imaging framework centered on CT descriptors was used to support consistent radiology–pathology correlations.

**Conclusions:**

These statements provide a unified taxonomy and diagnostic framework for appendiceal abscesses and related entities, improving interdisciplinary communication, and enabling cross-study comparability.

**Supplementary Information:**

The online version contains supplementary material available at 10.1007/s00384-026-05174-y.

## Introduction

Appendiceal abscesses, often grouped with appendiceal phlegmon, occur in a minority of acute appendicitis cases (~ 2–10%) and have important clinical consequences [1, 2]. However, terminology and diagnostic descriptors remain inconsistent across studies and clinical settings. This heterogeneity limits communication, reduces cross-study comparability, and contributes to variability in interpretation and downstream management. This lack of standardization hinders clinical communication, complicates cross-study comparability, and contributes to variability in diagnostic interpretation and management [3, 4].

Over recent decades, multiple definitions and classification schemes have been proposed that often reflect local practice patterns. Consequently, appendiceal abscesses are not consistently distinguished from related entities, such as appendiceal masses (phlegmon) or perforated appendicitis, leading to ambiguity in both clinical decision-making and research reporting. These limitations highlight the need for a shared, multidisciplinary conceptual framework spanning surgical, radiologic, and pathologic domains [1, 3–5]. In this context, the American Association for the Surgery of Trauma (AAST) Emergency General Surgery grading system provides an established severity taxonomy integrating clinical, imaging, operative, and pathological criteria, with demonstrated prognostic validity in appendicitis [6].

Diagnostic imaging is central to the identification and characterization of appendiceal abscesses. Ultrasound is frequently used as the initial modality, particularly in younger patients, whereas computed tomography (CT) offers the highest diagnostic accuracy for detecting complicated appendicitis and defining abscess morphology [7–9]. Nevertheless, imaging definitions and reporting criteria remain variable, with implications for diagnostic performance, interobserver agreement, and downstream clinical decisions [9].

To address these gaps, the Italian Society of Surgical Research (SIRC) and Italian Society of Emergency Surgery and Trauma (SICUT) convened a multidisciplinary expert panel to develop standardized, clinically applicable definitions and diagnostic descriptors for appendiceal abscesses. A structured, four-round modified Delphi was implemented, culminating in an in-person consensus conference held in Rome on November 6, 2025. Definitions and descriptors were aligned, where possible, with internationally recognized frameworks to enhance consistency and external validity.

Importantly, this initiative was designed to provide consensus-based definitions and an operational diagnostic framework rather than evidence-graded clinical recommendations. Where high-quality evidence is limited or heterogeneous, the proposed framework reflects multidisciplinary expert agreement aimed at improving conceptual clarity and real-world applicability.

## Methods

**Fig. 1:**
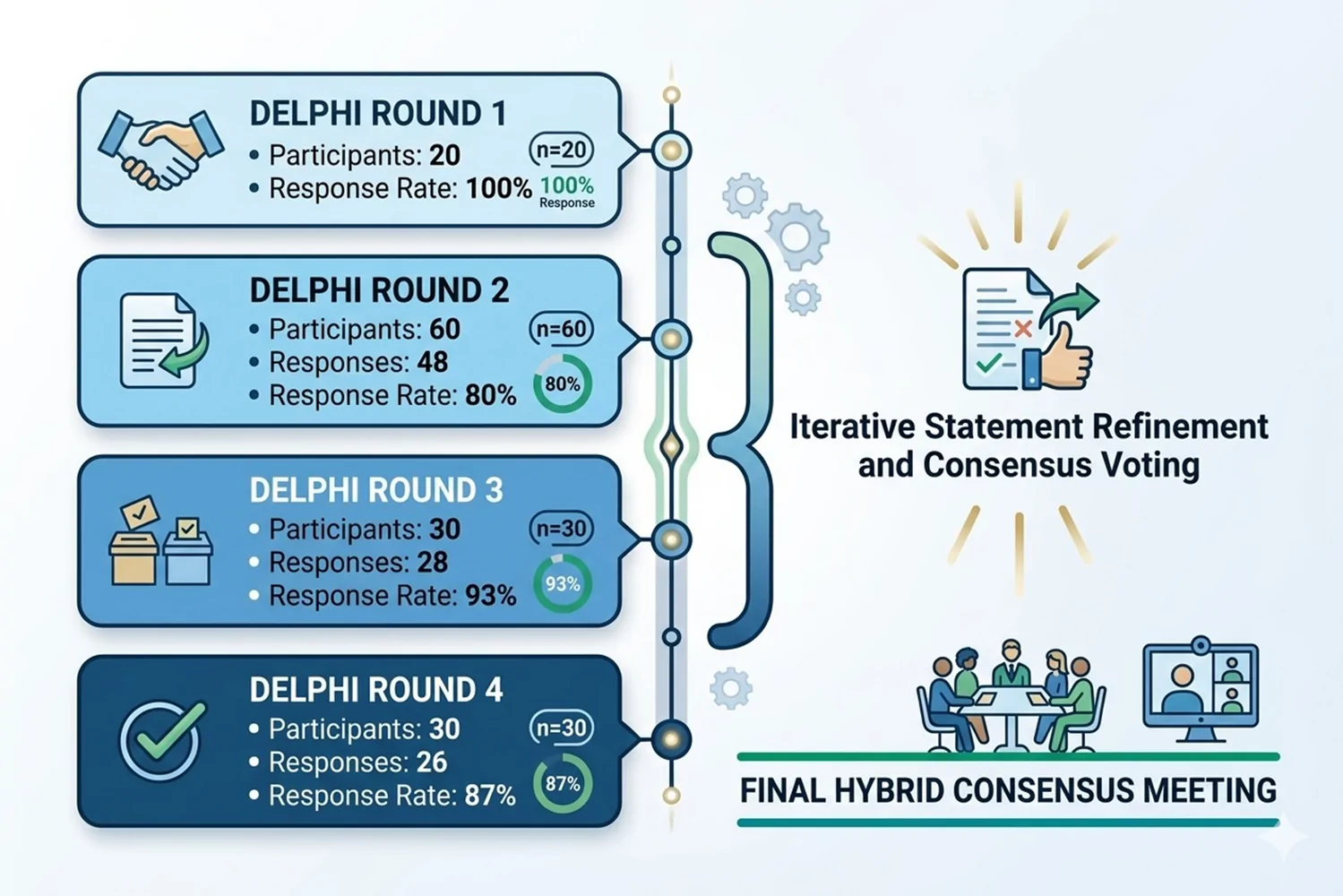
Modified Delphi process. Schematic representation of the four-round modified Delphi process used to develop consensus-based definitions and diagnostic descriptors for appendiceal abscess

A core working group developed the project protocol and governance framework, which was approved by the executive boards of both scientific societies before the study initiation. The project was conducted under the auspices of the SIRC and SICUT using a four-round modified Delphi, culminating in an in-person consensus conference (Rome, November 6, 2025). The modified Delphi process is summarized in Fig. 1. This manuscript reports the Part 1 statements (n = 7); the remaining statements from the overall project (total n = 22) are presented in companion manuscripts.

The panel formulated a single overarching question focused on standardizing definitions and diagnostic constructs for appendiceal abscesses. Management-related elements (clinical and laboratory assessment, imaging pathways, microbiology, and treatment options) were considered as contextual background and were not the primary objective of Part 1.

This project aimed to develop consensus-based definitions and an operational diagnostic framework. It was not designed to produce evidence-graded clinical recommendations. Accordingly, elements related to clinical management should be interpreted as contextual or hypothesis-generating rather than prescriptive recommendations.

### Literature search and evidence synthesis

A systematic literature search was conducted following PRISMA principles, where applicable, noting that a formal GRADE assessment was not performed. The purpose of the literature review was to inform the consensus process rather than to generate independent evidence-based recommendations and to provide a structured evidence base to support multidisciplinary expert discussion.

Databases included PubMed/MEDLINE and Scopus as primary sources, with Google Scholar was used as a supplementary database (first 200 results by relevance screened). Searches were updated on October 1, 2025. Google Scholar was used only to identify potentially missed records, acknowledging limited reproducibility due to its non-transparent ranking algorithm. Citation chasing (reference lists and related-article functions) was also performed.

Search strategies combined MeSH terms and free-text keywords related to appendiceal abscess, complicated appendicitis, appendiceal mass/phlegmon, intra-abdominal abscess, CT, ultrasound, percutaneous drainage, and antibiotic therapy.

Screening was conducted in two stages (title/abstract, then full text). Two independent reviewers assessed eligibility using predefined criteria, with disagreements resolved by discussion or third-party adjudication. When multiple overlapping studies were identified, the most recent or largest dataset was selected. Additional relevant studies were identified through reference list screening.

Eligible designs included randomized trials, cohort studies, case series, systematic reviews, and meta-analyses. Clinical questions were structured using the PICO framework to support focused evidence retrieval, recognizing that the resulting evidence was synthesized narratively and interpreted within the consensus process rather than used to derive quantitatively graded recommendations.

### Evidence appraisal

The available evidence was synthesized to inform the Delphi process. Given the objective of standardizing definitions and diagnostic descriptors in a conceptually heterogeneous field, the synthesis was conducted as a structured narrative review rather than a formal guideline process.

Although studies were systematically identified and framed using PICO, no formal evidence-grading system (e.g., GRADE) or risk-of-bias tools were applied, and the strength of evidence was not quantitatively assessed.

Accordingly, the evidence synthesis served to support expert discussion rather than generate graded recommendations. Evidence-derived findings and consensus-based interpretations were considered complementary but conceptually distinct components of the process.

The Delphi methodology enabled integration of available data with multidisciplinary expert judgment, particularly where evidence is limited or heterogeneous.

Where statements extend beyond the directly available evidence base, they reflect structured expert consensus intended to enhance interpretability and clinical applicability, rather than empirically validated recommendations.

As a result, statements should be interpreted as consensus-based definitions and diagnostic constructs rather than evidence-graded clinical recommendations. This approach is appropriate for areas with conceptual variability but requires future validation through formal evidence appraisal and external studies.

### Delphi methodology and panel

A four-round modified Delphi approach was used to achieve consensus on definitions, classification items, and diagnostic statements. The multidisciplinary panel included experts in emergency surgery, general surgery, radiology, pathology, infectious diseases, clinical methodology, and critical care. Selection criteria included expertise, scientific output, and geographic representation with the aim of ensuring a balanced multidisciplinary perspective to inform consensus-based outputs rather than evidence-derived recommendations.

- **Round 1 (electronic):** Twenty international experts independently reviewed and rated the initial draft statements and provided structured qualitative feedback via email. The individual ratings were anonymous. This exploratory round was primarily aimed at refining conceptual definitions and identifying areas of uncertainty rather than validating evidence-based recommendations.
- **Round 2 (electronic):** Sixty national experts completed a second anonymous electronic survey to evaluate revised statements. This phase aimed to assess clarity, applicability, and agreement on consensus-driven constructs across a broader panel.
- Round 3 (hybrid consensus meeting): A hybrid (in-person and online) meeting was held in Rome on November 6, 2025. Thirty experts discussed the unresolved statements and assessed their agreement using real-time anonymous electronic voting. The Wooclap platform enabled live interaction; speakers presented each statement and participants provided instant feedback via personal devices (smartphones, tablets, or laptops). Votes were categorized as “agree,” “neutral,” or “disagree” on a three-point Likert scale. Percentage agreement was immediately displayed. Statements were iteratively refined through structured discussion to achieve convergence of expert opinion.
- Round 4 (electronic): A final remote electronic round on December 29, 2025, was used to re-rate statements that had not met the predefined consensus threshold in previous rounds, with the objective of consolidating agreement on statements derived from the consensus process.

#### Consensus thresholds and statement handling

Predefined thresholds were applied: ≥ 80% agreement for acceptance and ≥ 95% for strong consensus. Agreement was calculated as the proportion of “agree” responses among all votes, with neutral responses included in the denominator but not the numerator. These thresholds were defined a priori to operationalize the level of agreement within the panel and do not represent gradings of evidence strength.

Statements not meeting consensus were revised and resubmitted. Persistent disagreements were prioritized for discussion during the consensus meeting, reflecting areas of conceptual uncertainty rather than discrepancies in high-certainty evidence.

### Oversight, conflicts of interest, and approvals

The process was conducted under SIRC and SICUT oversight and approved by both governing boards. All panel members declared conflicts of interest, which were managed according to institutional policies.

An independent external review by four non-panel experts was performed, assessing protocol, wording, and transparency. This external review focused on methodological rigor, clarity, and consistency between evidence synthesis and consensus-based statements. Feedback was incorporated where appropriate. Final statements were formally approved before dissemination.

### Data management and archiving

Likert responses (“agree,” “neutral,” “disagree”) were coded as 3, 2, and 1. Median and interquartile range (IQR) were calculated to describe agreement, providing a quantitative summary of consensus rather than a measure of evidence quality or strength.

Response rates were recorded across all Delphi rounds. In Round 1, all 20 invited international experts responded (100%). In Round 2, 48 of 60 invited participants completed the survey (80%). During the consensus meeting (Round 3), 28 of 30 invited experts participated in real-time voting (93%). In Round 4, 26 of 30 invited participants completed the final survey (87%).

The variation in panel size across rounds reflects the structured design of the modified Delphi process. Round 1 involved a limited number of highly selected international experts to define initial concepts and terminology. Round 2 expanded to a broader panel to test clarity, applicability, and generalizability of the statements. Round 3 consisted of a focused consensus meeting with selected participants to resolve outstanding disagreements through structured discussion and real-time voting. Round 4 was conducted to re-evaluate statements not reaching the predefined consensus threshold.

This staged approach is consistent with established Delphi methodology and allows both depth of expertise and broader validation, with the resulting metrics interpreted as indicators of agreement within the panel rather than as proxies for evidentiary certainty.

## Results

This initial set of consensus statements establishes standardized pathological definitions across the spectrum of acute appendicitis, with a specific focus on appendiceal abscesses. Uniform terminology was identified as essential to reduce diagnostic ambiguity, improve interdisciplinary communication, and enhance comparability across clinical studies.

**Fig. 2:**
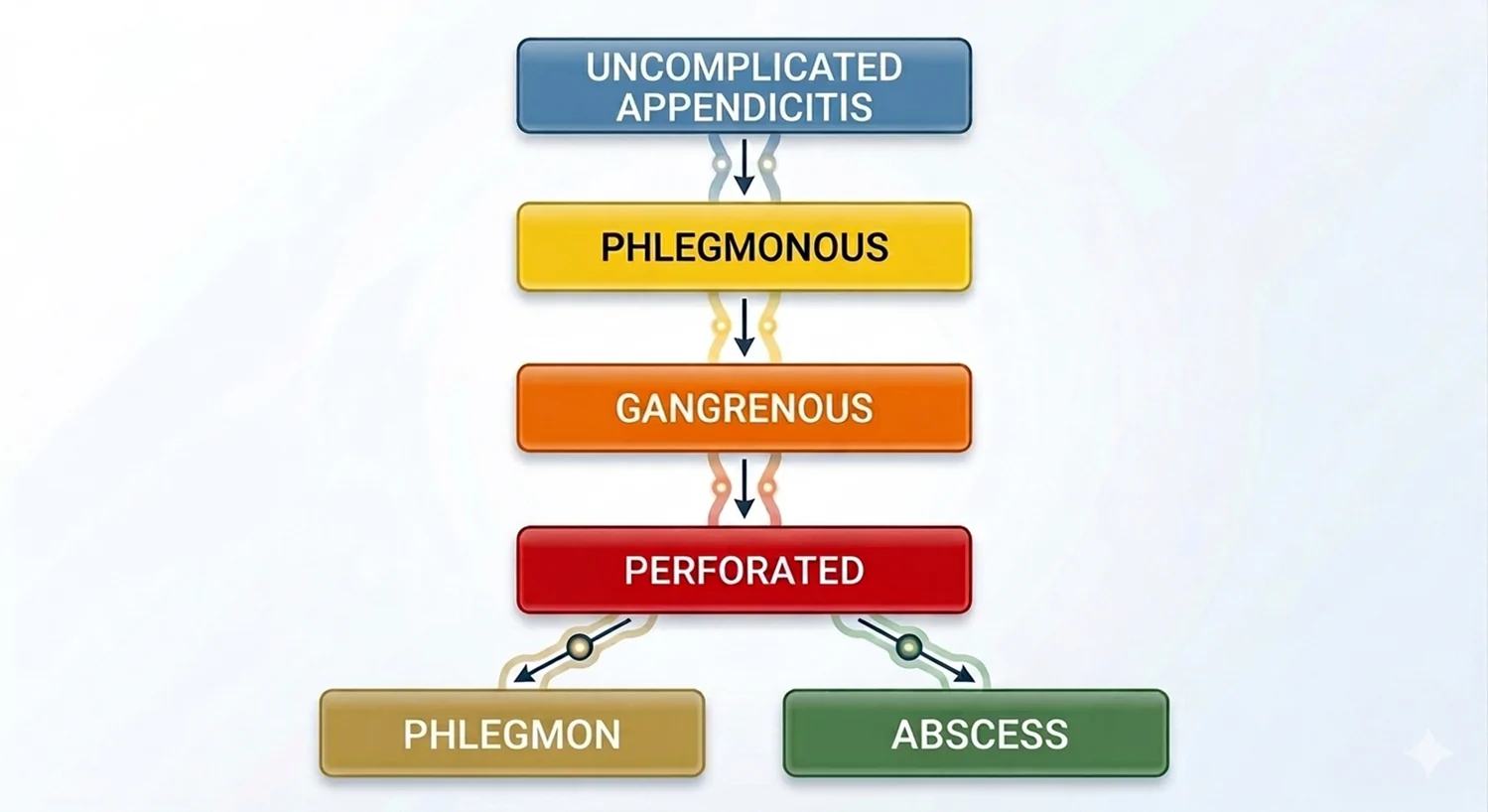
Pathological continuum and clinical–radiological outcomes in acute appendicitis

Seven statements (1.1–1.7) define key entities along the inflammatory spectrum, distinguishing uncomplicated from complicated appendicitis and providing clear definitions for phlegmonous, gangrenous, and perforated appendicitis, as well as appendiceal abscess and appendiceal mass (phlegmon). These definitions were aligned with established pathological criteria and current imaging correlates to support applicability in both clinical practice and research within a consensus-based framework rather than as evidence-graded diagnostic criteria (Fig. 2).

Response rates remained high across all rounds (80–100%), supporting the robustness of the consensus process (Supplementary Table S1). All statements achieved high levels of agreement, confirming broad consensus on a shared pathological framework as the basis for classification, imaging interpretation, and contextual clinical decision-making, where applicable.

The panel also endorsed a CT-centered imaging framework to support consistent radiology–pathology correlation and standardized reporting. This framework is intended as an operational aid derived from expert consensus and available evidence, rather than as a prescriptive diagnostic algorithm. Statements reaching unanimous agreement (1.1, 1.2, 1.5, 1.6) and near-unanimous agreement (1.3, 1.4, 1.7) showed a median score of 3 and an interquartile range of 0, indicating minimal variability among panelists (Supplementary Table S2).

Neutral responses were infrequent and did not materially influence the consensus outcomes.

Statement 1.1 — Uncomplicated Appendicitis (Agreement: 100%).

Uncomplicated appendicitis is defined as localized appendiceal inflammation without transmural ischemic necrosis or perforation, characterized by preservation of wall integrity and inflammatory infiltration limited to the mucosa and submucosa.

This definition excludes any form with mural disruption or extra-appendiceal extension, including gangrenous or perforated appendicitis, periappendiceal phlegmon, or abscess, ensuring a clear distinction between uncomplicated and complicated disease.

In clinical practice, imaging serves as a surrogate for pathological classification. On ultrasound or contrast-enhanced CT, uncomplicated appendicitis typically presents with appendiceal wall thickening and periappendiceal fat stranding in the absence of features suggestive of complication, such as rim-enhancing collections, extraluminal air, or focal mural defects [7, 9]. Although CT provides the highest diagnostic accuracy for this distinction, findings should be interpreted in conjunction with clinical and laboratory data.

A standardized definition may support risk stratification and clinical decision-making, including the identification of patients who may be considered candidates for nonoperative management within an integrated clinical assessment and in line with existing evidence and local practice. In cases of diagnostic uncertainty, particularly early in the disease course, reassessment incorporating clinical evolution, laboratory markers, and repeat or conditional imaging may be considered based on clinical judgment and within a consensus-informed framework.

Statement 1.2 — Complicated Appendicitis (Agreement: 100%).

Complicated appendicitis is defined as transmural inflammation extending through the full thickness of the appendiceal wall, with potential compromise of wall integrity or extension beyond the appendix. This category includes gangrenous appendicitis, perforation (free or contained), and localized consequences of contained perforation such as phlegmon (appendiceal mass) and abscess.

This definition establishes a clear distinction from uncomplicated appendicitis based on transmural involvement and extra-appendiceal extension.

From an imaging perspective, features suggestive of complicated appendicitis include rim-enhancing fluid collections, inflammatory masses (phlegmon), extraluminal gas, extraluminal appendicoliths, and focal wall defects [7, 9]. These findings support identification of advanced disease but should be interpreted in conjunction with clinical and laboratory data.

From a clinical standpoint, complicated appendicitis is generally associated with increased inflammatory burden and higher risk of adverse outcomes, which, based on available evidence and panel consensus, may justify closer monitoring and consideration of source-control strategies when clinically appropriate [3].

Diagnostic uncertainty may occur, particularly in early or partially treated disease; in such cases, classification should integrate clinical, laboratory, and imaging findings, with reassessment over time when clinically indicated and interpreted within a consensus-informed framework [5, 7, 9].

Statement 1.3 — Phlegmonous Appendicitis (Agreement: 98%).

Phlegmonous appendicitis is defined as a form of acute appendicitis characterized by transmural inflammatory involvement of the appendiceal wall without evidence of mural disruption or extra-appendiceal pus collection.

It represents an intermediate stage within the inflammatory spectrum, between uncomplicated disease and more advanced gangrenous or perforated forms.

From a pathological perspective, it is characterized by transmural neutrophilic infiltration with mural edema, in the absence of ischemic necrosis or wall discontinuity, although distinction from early gangrenous appendicitis may be challenging without histological confirmation [5, 10].

On imaging, phlegmonous appendicitis typically presents with marked wall thickening, mural enhancement, and periappendiceal fat stranding, without features of perforation such as extraluminal gas or abscess formation [5, 7].

Recognition of this entity may support disease stratification; however, its clinical implications remain variable, and it should be interpreted as a consensus-based descriptive construct rather than a distinct evidence-based management category.

Statement 1.4 — Gangrenous Appendicitis (Agreement: 98%).

Gangrenous appendicitis is defined as a form of acute appendicitis characterized by transmural ischemic necrosis of the appendiceal wall, typically associated with vascular compromise, in the absence of a demonstrable full-thickness wall defect.

It represents an advanced stage of disease, distinct from phlegmonous appendicitis (transmural inflammation without necrosis) and from perforated appendicitis, which is defined by a full-thickness wall defect.

From a pathological perspective, it is characterized by full-thickness ischemic necrosis with microscopic loss of mural integrity, often associated with vascular thrombosis and tissue friability [10].

On imaging, features suggestive of gangrenous appendicitis include reduced, heterogeneous, or absent mural enhancement on contrast-enhanced CT, reflecting impaired perfusion. Additional findings may include marked wall thickening and periappendiceal fat stranding, with occasional intramural gas [5, 11].

Recognition of gangrenous appendicitis may assist in assessing disease severity; however, differentiation from early perforation may be challenging, and classification should be integrated with clinical, laboratory, and imaging findings within an overall clinical assessment and interpreted in a consensus-informed context.

Statement 1.5 — Perforated Appendicitis (Agreement: 100%).

Perforated appendicitis is defined as a full-thickness defect of the appendiceal wall with communication between the lumen and the peritoneal cavity, resulting in spillage of luminal contents.

Depending on the degree of containment, perforation may manifest as localized peritonitis, phlegmon, abscess, or diffuse peritoneal involvement.

From a pathological perspective, perforation corresponds to a macroscopic or microscopic breach of wall integrity, although direct visualization is not always feasible in clinical practice, particularly in contained or sealed perforation [5, 11].

On contrast-enhanced CT, findings suggestive of perforation include focal wall discontinuity, extraluminal gas, periappendiceal fluid collections, or abscess formation [11, 12]. These features support the diagnosis but should be interpreted in conjunction with clinical and laboratory findings.

From a clinical standpoint, identification of perforation reflects advanced disease severity and may be associated with differences in management strategies, although decisions are based on an integrated assessment rather than a single imaging criterion. The distinction between free and contained perforation may contribute to treatment selection within an overall clinical assessment, but is not always reliably defined by imaging alone.

Statement 1.6 — Appendiceal Abscess (Agreement: 100%).

Appendiceal abscess is defined as a localized collection of purulent material arising from a contained perforation of the appendix, typically delimited by an inflammatory wall formed by adjacent tissues.

From a pathological perspective, it corresponds to a circumscribed cavity containing pus, surrounded by an inflammatory wall composed of fibrin, granulation tissue, and adjacent structures [5, 10]. This distinguishes appendiceal abscess from appendiceal mass (phlegmon), which is characterized by a predominantly solid inflammatory conglomerate without a well-formed fluid collection.

On contrast-enhanced CT, appendiceal abscess typically appears as a well-defined, rim-enhancing fluid collection with surrounding inflammatory changes. Additional features may include extraluminal gas, reactive bowel wall thickening, or mass effect on adjacent structures [13–21].

From a clinical perspective, identification of an abscess reflects contained perforation and may support therapeutic planning, including consideration of image-guided drainage and antibiotic therapy where appropriate. Management decisions, however, should be individualized and based on the overall clinical and radiologic assessment.

Statement 1.7 — Appendiceal Mass (Phlegmon) (Agreement: 98%).

Appendiceal mass (phlegmon) is defined as an inflammatory conglomerate composed of the inflamed appendix and adjacent structures, including omentum, bowel loops, and mesenteric fat, resulting from localized containment of the inflammatory process. On imaging, it appears as an ill-defined soft-tissue mass without a well-demarcated, rim-enhancing fluid collection.

From a pathological perspective, it represents a predominantly solid inflammatory process extending beyond the appendix [22], in contrast to appendiceal abscess, which is characterized by a localized purulent collection with a defined capsule [5, 10].

On contrast-enhanced CT, an appendiceal mass typically presents as an ill-defined inflammatory area with soft-tissue density, marked periappendiceal fat stranding, and involvement of adjacent bowel loops [5, 13]. The absence of a rim-enhancing fluid collection supports differentiation from abscess, although overlap may occur, particularly in early or evolving stages.

### Operational use of imaging modalities across clinical scenarios

Although these entities are defined primarily on histopathology, cross-sectional imaging plays a central role in real-time diagnosis, grading, and clinical triage. Imaging therefore acts as the operational surrogate for pathology in routine practice.

In nonpregnant adults, contrast-enhanced CT provides the highest overall accuracy for differentiating uncomplicated from complicated appendicitis and for characterizing abscess morphology and extent. In pediatrics and pregnancy, ultrasound is typically first-line; when ultrasound is nondiagnostic, MRI is preferred in pregnancy, while in children second-line imaging (MRI or CT) may be guided by local pathways and resource availability.

While contrast-enhanced CT represents the reference standard in nonpregnant adults, the selection of imaging modality should be adapted to patient characteristics and clinical context.

In pediatric patients, ultrasound is generally considered the first-line imaging modality due to its safety profile and lack of ionizing radiation. When ultrasound findings are nondiagnostic or inconclusive, second-line imaging (MRI or CT) may be guided by local expertise and availability, with MRI often preferred where accessible.

In pregnant patients, ultrasound is typically used as the initial modality of choice. In cases of nondiagnostic ultrasound, MRI may be considered as the preferred second-line technique, given its high diagnostic accuracy and absence of ionizing radiation. CT may be reserved for selected cases in which both ultrasound and MRI are inconclusive and diagnostic uncertainty persists.

In nonpregnant adults, CT remains the primary imaging modality due to its superior diagnostic accuracy in differentiating uncomplicated from complicated appendicitis and in characterizing abscess morphology.

To improve reproducibility, Table 1 summarizes typical CT and ultrasound features distinguishing appendiceal abscess (well-defined, rim-enhancing fluid collection) from appendiceal mass/phlegmon (ill-defined, predominantly solid inflammatory conglomerate without a mature capsule). These descriptors are intended as pattern-based aids rather than strict diagnostic criteria, as radiology–pathology concordance is not absolute, particularly in early or partially treated disease.

**Table 1:** Typical CT and ultrasound imaging features of appendiceal abscess and appendiceal mass (phlegmon)

Imaging feature	Appendiceal abscess (CT)	Appendiceal abscess (US)	Appendiceal mass/phlegmon (CT)	Appendiceal mass/phlegmon (US)
Rim-enhancing fluid collection	+ + +	Anechoic/hypoechoic collection with vascularized wall on Doppler: + + +	−/+	−/+ (poorly demarcated, no true capsule)
Well-defined capsule	+ + +	+ + + (hyperemic rim)	−	−
Internal/extraluminal gas	+/−	Echogenic foci with dirty shadowing or ring-down: +/−	−	−
Predominantly solid inflammatory tissue	−	−	+ + +	+ + + (echogenic, ill-defined, tethering of adjacent loops)
Ill-defined inflammatory tissue/conglomerate	−/+	−/+	+ + +	+ + +
Focal appendiceal wall defect (mural discontinuity)	+/− (when visible)	– (not reliably assessed)	−	−
Extraluminal appendicolith	+/−	−/+ (echogenic focus with posterior shadowing)	−	−
Periappendiceal fat stranding/inflammatory changes	+ + +	Correlate: hyperechoic mesenteric fat, tender on probe: + + +	+ + +	+ + +
Reactive thickening of adjacent bowel loops	+/−	+/−	+/−	+/−
Mass effect on cecum/ileum	+/−	+/−	+/−	+/−
Color Doppler peripheral hyperemia	N/A	+ + + (capsule/periphery)	N/A	+ + (peripheral rather than central)
Posterior acoustic enhancement	N/A	+/− (when fluid content predominates)	−	−
Drainable collection (image-guided feasibility)	Yes	Yes (when mature and safely accessible)	No	No

+ + + = typical/frequent; −/+ = possible; − = typically absent; +/− = variable; N/A = not applicable

Gray zones may occur, especially between evolving abscess and phlegmon. In equivocal cases, short-interval clinical reassessment (typically within 6–12 h) integrating symptoms, vital signs, and inflammatory markers (e.g., WBC, CRP) may be considered within an overall clinical assessment. Repeat imaging within 12–24 h may be considered when clinically appropriate, particularly if clinical or laboratory findings fail to improve or suggest progression, with modality selection guided by patient group and local protocols.

Descriptors are intended as typical patterns to support radiology–pathology correlation; they were derived from the final consensus statements and literature synthesis rather than voted as standalone Delphi items.

We have also included a concise operational rule summarizing key CT findings associated with complicated appendicitis to further enhance clinical applicability and bedside usability (Table 2), which should be interpreted as a consensus-based aid rather than a prescriptive diagnostic algorithm.

**Table 2:** Practical diagnostic approach (suggested operational rule)

Complicated appendicitis should be suspected when ≥ 1 of the following CT findings is present: – Extraluminal gas – Rim-enhancing fluid collection – Focal wall discontinuity – Extraluminal appendicolith
In the absence of these features, isolated appendiceal wall thickening with periappendiceal fat stranding suggests uncomplicated disease

### Operational diagnostic considerations and interobserver variability

Although the proposed definitions are rooted in histopathology, their application in clinical practice relies predominantly on imaging and intraoperative assessment. Concepts such as “transmural inflammation” or “localized containment” cannot always be directly visualized and may therefore be interpreted using reproducible surrogate markers.

From a radiological perspective, differentiation between uncomplicated and complicated appendicitis is generally based on a combination of practical CT findings rather than a single feature. Findings most consistently associated with complicated disease include: (i) extraluminal gas, (ii) rim-enhancing fluid collections, (iii) extraluminal appendicolith, and (iv) focal wall discontinuity. Conversely, the absence of these findings, in the presence of isolated wall thickening and periappendiceal fat stranding, may support a diagnosis of uncomplicated disease.

The distinction between appendiceal abscess and appendiceal mass (phlegmon) is likewise based on pattern recognition rather than strict criteria. A well-defined, rim-enhancing fluid collection is typically considered indicative of abscess, whereas a predominantly solid, ill-defined inflammatory mass without a capsule generally favors phlegmon. However, overlap may occur, particularly in evolving or partially treated disease.

Interobserver variability remains an inherent limitation, especially in borderline cases such as early perforation or evolving abscess formation. For this reason, the proposed framework is intended to be applied as a probabilistic classification system rather than a binary taxonomy.

In equivocal scenarios, short-interval reassessment integrating clinical evolution, laboratory markers (e.g., WBC, CRP), and repeat imaging (12–24 h) may be considered within an overall clinical assessment to improve diagnostic confidence and reduce misclassification.

### International applicability and external validity of the consensus

Although the consensus was coordinated by two Italian scientific societies, methodological safeguards were implemented to enhance international applicability and minimize geographic or practice-related bias. The initial Delphi round included international experts, and the use of anonymous scoring, iterative feedback, and structured revisions supported convergence toward broadly shared terminology.

To ensure semantic and taxonomic consistency, the panel aligned pathological definitions and imaging descriptors with major international frameworks, including the EAES consensus, WSES Jerusalem guidelines, the AAST Emergency General Surgery grading system, and the imaging–pathology classification by Hoffmann et al. This alignment is intended to facilitate comparability with the global literature and may support adoption across diverse healthcare systems [3–5, 15].

The proposed definitions are intended to be relatively independent of local resources, imaging protocols, and surgical practices, thereby potentially supporting applicability in both high- and resource-limited settings. Further multinational validation studies are warranted to assess reproducibility and interobserver agreement across different clinical contexts [3, 4].

Finally, the AAST grading system, which integrates clinical, imaging, operative, and pathological criteria, provides an externally validated, outcome-oriented framework that supports the potential generalizability of the present taxonomy [6].

### Limitations

Part 1 establishes a standardized framework for pathological definitions, classification, and imaging-based diagnosis across the spectrum of acute appendicitis, with specific emphasis on appendiceal abscess and appendiceal mass (phlegmon). Alignment with major international classifications is intended to support consistent adoption across healthcare systems [3, 4, 6].

This work represents the first part of a two-part project, with Part 1 focusing on definitions, taxonomy, and diagnostic pathways, and Part 2 addressing therapeutic strategies and clinical management.

Several limitations should be acknowledged. By reducing heterogeneity in terminology and imaging interpretation, the proposed framework is intended to provide clear and reproducible definitions spanning uncomplicated and complicated appendicitis, although its impact on clinical outcomes and decision-making has not been formally evaluated [3, 4].

The explicit differentiation between phlegmonous, gangrenous, and perforated appendicitis, as well as between abscess and appendiceal mass, may enhance radiology–surgery communication and may support more precise assessment of disease severity, but these effects remain to be validated in prospective studies [3, 5].

Importantly, some of the operational and clinical implications discussed in this manuscript extend beyond the directly available evidence base and reflect structured expert consensus aimed at improving interpretability and applicability in clinical practice. These elements should therefore be interpreted cautiously and not as evidence-graded recommendations.

Finally, integration with the AAST Emergency General Surgery grading system provides an externally validated, outcome-oriented reference framework, which may support the external consistency of the proposed taxonomy but does not substitute for independent validation of the definitions introduced in this consensus [6].

A further limitation relates to the geographic composition of the panel. Although international experts participated, the consensus process remained predominantly Italian and European, which may limit generalizability to non-European healthcare settings and underscores the need for external validation in broader international contexts.

## Conclusion

This first part of the SIRC/SICUT consensus provides a standardized, multidisciplinary framework for the definition, classification, and imaging-based characterization of appendiceal abscess and related entities across the spectrum of acute appendicitis. By harmonizing pathological, radiological, and clinical terminology, the framework aims to improve communication, reduce diagnostic variability, and enhance comparability across studies and clinical settings.

A key contribution is the explicit differentiation between uncomplicated and complicated appendicitis and the refined characterization of intermediate entities, including phlegmonous and gangrenous appendicitis, as well as the distinction between appendiceal abscess and appendiceal mass (phlegmon).

The proposed CT-centered framework and operational descriptors are intended as practical tools to support real-world diagnostic assessment. However, they should be interpreted as consensus-based constructs rather than evidence-graded diagnostic criteria or prescriptive clinical algorithms.

Although aligned with major international classifications, external validation is required. Some operational implications remain hypothesis-generating. Overall, this work establishes a shared conceptual and diagnostic foundation for appendiceal abscess and supports future research and standardization.

## Supplementary Information

Below is the link to the electronic supplementary material.

Supplementary file1 (DOCX 15 KB)

## Data Availability

No datasets were generated or analysed during the current study.

## Declarations

**Ethical approval**

Not applicable. This is an expert consensus based on published literature and panel voting; no patient-level data were collected.
