# Supplementary material for: Appendiceal abscess: pathologic definitions and a diagnostic framework—SIRC/SICUT consensus
Source: Int J Colorectal Dis. 2026 Jun 15;41(1):139. doi: 10.1007/s00384-026-05174-y (PMC13500402; doi:10.1007/s00384-026-05174-y)
Supplement: Supplementary file 1 [file 384_2026_5174_MOESM1_ESM.docx]

Supplementary Table S1. Delphi voting results and quantitative agreement metrics

| **Round** | **Type of round** | **Invited (n)** | **Respondents (n)** | **Response rate (%)** |
| --- | --- | --- | --- | --- |
| Round 1 | Electronic  (international panel) | 20 | 20 | 100 |
| Round 2 | Electronic  (expanded panel) | 60 | 48 | 80 |
| Round 3 | Consensus meeting (hybrid) | 30 | 28 | 93 |
| Round 4 | Electronic  (final validation) | 30 | 26 | 87 |

Supplementary Table S2 – Delphi voting results

| **Statement** | **Agree (%)** | **Neutral (%)** | **Disagree (%)** | **Median** | **IQR** |
| --- | --- | --- | --- | --- | --- |
| 1.1 | 100 | 0 | 0 | 3 | 0 |
| 1.2 | 100 | 0 | 0 | 3 | 0 |
| 1.3 | 98 | 2 | 0 | 3 | 0 |
| 1.4 | 98 | 2 | 0 | 3 | 0 |
| 1.5 | 100 | 0 | 0 | 3 | 0 |
| 1.6 | 100 | 0 | 0 | 3 | 0 |
| 1.7 | 98 | 2 | 0 | 3 | 0 |
